# Stem cell models of polyglutamine diseases and their use in cell-based therapies

**DOI:** 10.3389/fnins.2015.00247

**Published:** 2015-07-14

**Authors:** Evangelia K. Siska, George Koliakos, Spyros Petrakis

**Affiliations:** ^1^Biohellenika Biotechnology CompanyThessaloniki, Greece; ^2^Laboratory of Biochemistry, AHEPA University Hospital, Medical School, Aristotle University of ThessalonikiThessaloniki, Greece

**Keywords:** polyglutamine, neurodegeneration, mesenchymal stem cells, cell model, transplantation

## Abstract

Polyglutamine diseases are fatal neurological disorders that affect the central nervous system. They are caused by mutations in disease genes that contain CAG trinucleotide expansions in their coding regions. These mutations are translated into expanded glutamine chains in pathological proteins. Mutant proteins induce cytotoxicity, form intranuclear aggregates and cause neuronal cell death in specific brain regions. At the moment there is no cure for these diseases and only symptomatic treatments are available. Here, we discuss novel therapeutic approaches that aim in neuronal cell replacement using induced pluripotent or adult stem cells. Additionally, we present the beneficial effect of genetically engineered mesenchymal stem cells and their use as disease models or RNAi/gene delivery vehicles. In combination with their paracrine and cell-trophic properties, such cells may prove useful for the development of novel therapies against polyglutamine diseases.

## Polyglutamine diseases

Polyglutamine (polyQ) diseases are late-onset progressive neurodegenerative disorders; they include Huntington's disease (HD), Dentatorubral-pallidoluysian atrophy (DRPLA), Spinal and bulbar muscular atrophy (SBMA), and Spinocerebellar ataxia (SCA) −1, −2, −3, −6, −7, and −17. They are caused by a trinucleotide (CAG) repeat expansion within the coding region of unrelated genes that do not seem to share a common function or conformation. With the exception of SBMA, mutant genes are inherited in an autosomal dominant manner. CAG expansions encode for longer polyQ chains in the produced protein (Orr, 2012). As a result, the mutant protein adopts a different conformation than the wild type protein which is accompanied by a loss of its functionality. A striking feature of the polyQ protein is its ability to form toxic species and misfold into oligomers that slowly aggregate into inclusions detected in the nucleus. PolyQ expansions in mutant proteins may be deleterious because they interfere with normal protein-protein interactions or mediate aberrant ones (Lam et al., 2006; Lim et al., 2008), resulting in loss or gain of function, respectively. Protein accumulation in the nucleus may also perturb the structure of protein complexes, leading to transcriptional changes and contributing to toxicity in neurons. This hypothesis is supported by the recruitment of transcription factors into polyQ protein aggregates (Shimohata et al., 2000). Despite wide expression of mutant genes, formation of aggregates is accompanied by selective neurodegeneration. For example, in the case of SCA1 neuronal loss is mostly obvious in the Purkinje cell (PC) layer of the cerebellum (Orr and Zoghbi, 2001).

The symptoms of the disease manifest around the fourth decade of the life depending on the length of the polyQ tract in mutant proteins. The protein degradation machinery plays a pivotal role in the cellular response to expanded polyQ chains (Cummings et al., 1999; Bence et al., 2001). Chaperones bind to the misfolded proteins but fail to prevent their aggregation (Schmidt et al., 2002). Ubiquitinated forms of polyQ proteins are persistently associated with proteasome components indicating that cells are attempting to destroy misfolded proteins and that these substrates may be resistant to proteasomal degradation (Schmidt et al., 2002). Experimental evidence indicate that polyQ aggregates increase oxidative stress which may be responsible for the inhibition of proteasome activity (Diaz-Hernandez et al., 2006). In contrast, overexpression of proteins participating in the ubiquitin-proteasome complex protects cells and transgenic mice from polyQ-induced degeneration (Torashima et al., 2008; Petrakis et al., 2012b). These findings indicate that polyQ diseases are associated with a gradual inability of the cellular protein degradation machinery to remove misfolded proteins.

## Stem cell models of polyQ diseases

Cell models resembling the features of polyQ diseases are necessary in order to define the exact mechanisms of neuronal degeneration. Such models should present polyQ-induced cytotoxicity and intranuclear protein aggregation in a measurable manner and could be used for the identification of small molecules that would reverse these biochemical procedures. Immortalized laboratory cell lines as COS-1 or HEK have been extensively used for such purposes. However, they may not represent ideal models as they have evolved high chaperone activity under selective pressure in culturing conditions.

Induced pluripotent stem cells (iPSCs) have recently emerged as excellent tools for cell-based assays since they can differentiate into neuronal cell populations. Using fibroblasts from polyQ-disease patients, researchers have generated iPSC-derived neurons for SCA2, SCA3, HD, and SBMA (Koch et al., 2011; Camnasio et al., 2012; Xia et al., 2012; Nihei et al., 2013). These cells exhibit disease-associated changes in electrophysiology and metabolism due to polyQ expansions of the mutant proteins (HD iPSC Consortium, 2012). The iPSC technology holds great promise for cell biology and drug discovery. Researchers are expected to overcome current limitations in cell generation and proliferation. They will be also able to solve problems associated with genetic heterogeneity of fibroblasts obtained from different patients and carefully select cell lines with little risk for tumor formation. However, iPSC-derived neurons may not express a robust disease-associated phenotype, such as protein aggregates, that would facilitate their use in large high-throughput assays.

Mesenchymal stem cells (MSCs) represent a complementary alternative to iPSCs in terms of disease modeling. These adult stem cells can be easily isolated from various tissues (e.g., Wharton's jelly or adipose tissue) and expanded *ex-vivo* in large numbers (Pittenger et al., 1999). MSCs have a differentiation potential toward mesodermal cell lineages, even though under specific culturing conditions, they adopt characteristics of cells belonging to other germ layers (Kuroda et al., 2010). Recently, Dossena et al. (2014) showed that MSCs isolated from the adipose tissue of SBMA patients, partially form nuclear polyQ inclusions upon chemical inhibition of the proteasome. These findings suggest that a decline in proteasomal activity favors the gradual deposition of polyQ proteins in the nucleus.

**Figure 1:**
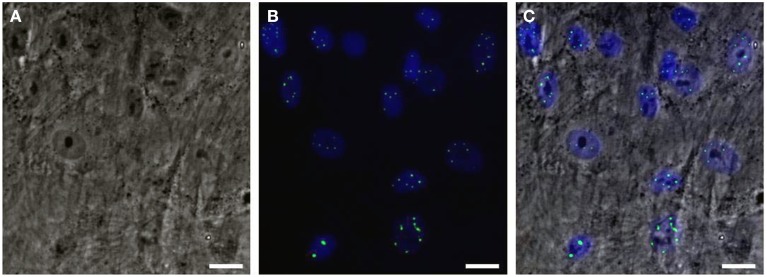
**Human mesenchymal stem cells overexpressing YFP-ATXN1Q82. (A)** Optical microscopy (brightfield) **(B)** Fluorescence microscopy. YFP-ATXN1Q82 protein aggregates are shown in green. Nuclei are stained with DAPI (blue), **(C)** Merged image (Scale bar = 25 μM).

Would it be possible to produce a robust pathogenic polyQ phenotype in MSCs? To this end, we generated an inducible mesenchymal stem cell model for the polyQ disease SCA1 using the Sleeping Beauty transposon system (Petrakis et al., 2012a). These cells stably overexpress the mutant gene ataxin-1 (ATXN1Q82) in fusion with Yellow Fluorescent Protein (YFP) under the Tet-On promoter. We expected that overproduction of the polyQ protein would result in a clear and measurable phenotype, such as large intranuclear protein aggregates. YFP-ATXN1Q82 MSCs stably express the mutant protein and accumulate large YFP-positive protein aggregates in the nucleus (Figure 1). YFP-ATXN1Q82 MSCs seem to retain their stem cell properties, such as fibroblastic morphology, self-renewal, proliferative capacity and expression of MSC surface markers (data not shown). The differentiation of YFP-ATXN1Q82 MSCs toward neuron-like cells would generate a population with a robust neuronal aggregation phenotype.

The efficient neuronal differentiation of MSCs is a matter of debate. Available protocols use chemical substances, cytokines or a combination of them to induce the formation of neuron-like cells. However, in several cases the obtained phenotypes are subtle and differentiated MSCs have limited electrophysiological properties and functionality. Therefore, breaking the barrier of the mesenchymal cell fate and directing MSCs toward an ectodermal cell lineage, would be a necessary step for efficient neurogenic differentiation of MSCs.

Adherent cultures of MSCs spontaneously form rare cell clusters similar to those formed by human embryonic cells (ES). This indicates that a subpopulation of MSCs may have embryonic characteristics and multipotential differentiation properties. Recently, researchers have identified an MSC population which is resistant to severe cellular stress conditions (Kuroda et al., 2010). These cells termed as multilineage differentiating stress enduring (MUSE) cells can be isolated by FACS analysis since they express both the mesenchymal CD105 and the embryonic SSEA3 markers (Kuroda et al., 2013). MUSE cells also express a variety of pluripotency markers and can efficiently differentiate into ectodermal and endodermal cell lineages (Heneidi et al., 2013). We propose that MUSE-derived YFP-ATXN1Q82 MSCs differentiated toward neuronal cells, would be an excellent cell model for SCA1. Given their high proliferative capacity, these cells could be used for the identification of novel drugs in cell-based assays, which should be further validated in iPSC-derived neuronal models.

## Stem cell-based therapies of polyQ diseases

Development of therapies for polyQ diseases focuses mainly on the identifiaction of novel drugs through compound screening. Potential targets for such screenings include chaperones, caspases and polyglutamine proteins themselves. Several drugs have been used so far in clinical trials. However, the lack of a clear improvement of the disease symptoms underlines the importance for the development of novel effective therapies.

Novel therapeutic approaches focus on the development of cell replacement therapies for polyQ diseases. Such therapies aim in either replacing damaged neurons or activating the endogenous neurogenesis mechanisms of the brain. Stem cells represent a promising tool for regenerative medicine and the treatment of human disorders. In the following sections, we are going to present stem-cell transplantation attempts in models of polyQ diseases focusing on those using MSCs.

In the case of HD, medium spiny neurons (MSNs) of the striatum are selectively lost. Attempts to replace these neurons have been made using fetal striatal tissue or striatal progenitors derived from human ES cells (Aubry et al., 2008). Although, these attempts showed promising results in rodent models of striatal neuronal loss, similar transplantations in clinical trials failed to significantly mitigate the symptoms of the disease. Grafted cells showed little integration in the affected regions of the brain (Benraiss and Goldman, 2011). Since these attempts involved allogeneic transplantation to patients, several issues need to be reconsidered. A key point is the establishment of an appropriate immunosuppression protocol to efficiently avoid graft rejection.

Transplantation of neurons derived from iPSCs is another option for cell replacement strategies. Such cells could be used for autologous transplantations since they are generated by the patient's own fibroblasts. However, there are concerns that need to be taken into account, including the need for genetic correction of the polyQ gene in generated neurons. A recent study reported the generation of genetically corrected iPSCs from HD patients. iPSC-derived neural stem cells showed reversed disease phenotypes and striatal differentiation *in vivo* (An et al., 2012), indicating their potential therapeutic value. At the moment, there is a limited number of studies evaluating the effect of iPSC transplantation in animal models of polyQ diseases. Those to follow would have to deal with the problem of efficient cell integration into host neuronal circuitry. Despite that, iPSCs represent an ideal source of patient-specific neurons for cell replacement therapies.

A different approach focuses on the use of adult stem cells for restoration of normal brain function in polyQ diseases. Chintawar et al. evaluated the effect of neural precursor cells (NPCs) in a SCA1 mouse model that displays progressive ataxia and PC loss. NPCs were stereotactically engrafted into the cerebellum of SCA1 mice which showed improved motor skills after transplantation. Transplanted NPCs were detected in close vicinity to the molecular layer of PCs suggesting that their neuroprotective effect was mediated by direct contact with host neurons (Chintawar et al., 2009). Furthermore, NPCs were shown to functionally integrate into the host neural circuitry and to form gap junctions that mediate neuroprotection (Jaderstad et al., 2010). Interestingly, the effect of NPCs was more prominent in older animals suggesting that these cells may be protective mainly in mice with significant cell loss.

Other adult stem cell populations, such as MSCs, also exert neuroprotective effects in rodent models of neurodegenerative diseases. MSCs have the advantage of high proliferative capacity and low expression of major histocompatibility complex antigens (Pittenger et al., 1999). Thus, they could be used for allogeneic transplantations with minimum risk for graft rejection. In the polyQ field, MSCs have been used in several studies summarized in Table 1. Transplantation of MSCs in the brain has raised concerns about the safety of the procedure. These concerns are based on the assumption that MSCs are not of neuronal origin and may form tumor-like cell clusters when grafted in a neuronal environment. However, recent work showed that MSCs are normally present in neuronal tissue and are detected in the vascular niche of the adult human brain (Paul et al., 2012). Their role is not clear but they may support neovascularization in the case of brain injury.

**Table 1:** **Effect of MSCs transplantation in polyQ disease models**.

**Type of cells**	**Number of cells**	**Administration route**	**Disease model**	**Observations**	**References**
BM-MSCs	0.1 × 10^6^	Transplantation into the striatum	HD – N171^82Q^ mice	Decrease of striatum atrophy Proliferation and differentiation of endogenous neural stem cells	Snyder et al., 2010
BM-MSCs	0.2 × 10^6^	Transplantation into the striatum	HD – R6/2-J2 mice	Neuronal differentiation of MSCs Migration of MSCs to the damaged area Increased levels of chemokines and angiogenesis markers	Lin et al., 2011
NTF-MSCs	0.1 × 10^6^	Transplantation into the striatum	HD – R6/2-J2 mice	Temporary improvement in motor function Extension of transgenic mice life span	Sadan et al., 2012a
BM-MSCs	0.4 × 10^6^	Transplantation into the striatum	HD – HD 51 CAG rats	Long-term behavioral benefits	Rossignol et al., 2013
BM-MSCs	0.1 × 10^6^	Transplantation into the striatum	QA striatal lesioned rats	Increased levels of stem cell factor protein	Bantubungi et al., 2008
BM-MSCs	2 × 10^6^	Intravenous injection	QA striatal lesioned rats	Reduction of behavioral abnormalities	Edalatmanesh et al., 2009
BM-MSCs	0.25 × 10^6^	Transplantation into the cerebellum	Cerebellar ataxia – Lurcher mice	Expression of neurotrophic factors by MSCs Increased number of Purkinje cells Fusion with Purkinje cells Improvement of motor behavior	Jones et al., 2010
UC-MSCs	Repeated doses of 2 × 10^6^ cells/week	Intravenous injection	Cerebellar ataxia – ICR mice injected with Ara-C	Increased levels of neurotrophic factors Improvement of motor function in ataxic mice	Zhang et al., 2011
BM-MSCs	0.6 × 10^6^	Intrathecal injection	SCA1 – B05 line (Q82) mice	Mitigation of cerebellar neuronal disorganization Suppression of PC dendrites atrophy Improvement of motor behavior	Matsuura et al., 2013
BM-MSCs	42 × 10^6^ cells/ kg of body weight	Intravenous injection	SCA2- C57BL/6J SCA2 mice	Neuroprotective effect on cerebellar PCs	Chang et al., 2011
BM-MSCs overexpressing BDNF or NGF	0.3 × 10^6^	Transplantation into the striatum	HD – YAC 128 mice	Overexpression of neurotrophins by MSCs Improvement of motor behavior	Dey et al., 2010
BM-MSCs overexpressing DnaJB4 or Pcbp3	0.05 × 10^6^	Injection to right retro-orbital sinus	SCA1 – SCA1154Q/2Q mice	Fusion with Purkinje cells Decreased number of nuclear inclusions Increased number of surviving PCs	Chen et al., 2011

*Table shows the type and number of cells that were used in each experiment (BM, bone marrow; UC, umbilical cord; NTF, neurotrophic factors-secreting). It also shows the administration route, the disease model and the outcome of the assay as described in the relevant publication*.

When transplanted into the cerebellum of Lurcher mice, a model of cerebellar ataxia, MSCs rescued Purkinje cells. Treated mice also presented significant improvement in their motor behavior. Histological analysis indicated that donor cells had migrated throughout the cerebellum. Many of them were located adjacent to the PC layer and expressed neurotrophic factors. Also, a significant increase in the number of PCs was observed. These results suggest that MSCs migrate toward areas where neurodegenerative processes occur and may rescue degenerating cells through cell-trophic effects (Jones et al., 2010).

Using a SCA1 mouse model, Matsuura et al. (2013) showed that intrathecal injection of MSCs could tone down the cerebellar neuronal disorganization, suppress the atrophy of PC dendrites and normalize motor coordination deficits in mice. More importantly, two studies performed in an HD model showed that MSCs survived for several weeks after transplantation into the striatum, partially differentiated into neuronal cell populations and activated endogenous neurogenesis mechanisms. Increased levels of neurotrophins were detected in parallel with a migration of endogenous neural stem cells to the lesioned areas (Snyder et al., 2010; Lin et al., 2011). These findings indicate that transplanted MSCs may reduce striatal damage in HD through the release of neurotrophic factors. This conclusion is further supported by experiments with neurotrophic factors-secreting MSCs (NTF-MSCs) which produce high levels of BDNF, NGF, and VEGF. Using the quinolinic acid (QA) lesion model of HD, Sadan et al. (2012b) showed that NTF-MSCs migrated toward the lesioned striatum and attenuated QA excitotoxicity.

Several lines of evidence indicate that transplantation of MSCs in the brain promotes synaptic transmission of neuronal networks and regeneration of the Purkinje cell layer. These events may be attributed to direct communication of MSCs with damaged neurons and delivery of trophic factors through exosomes, gap junctions or tunneling nanotubules (Nakamura et al., 2014). In several cases, MSCs were observed to fuse with damaged PCs *in vivo*, an event monitored by the formation of heterokaryons (Bae et al., 2007). These heterokaryons display PC identities and have similar morphology and electrophysiological properties (Weimann et al., 2003; Johansson et al., 2008; Kemp et al., 2011). Such events are rare under normal conditions. However, they are markedly increased in the presence of inflammatory cytokines within a damaged tissue. MSCs have an earlier effect in promoting neuronal survival compared to NPCs (Matsuura et al., 2013). This observation may be partially explained by their fusion with damaged neurons. It also suggests that these events are physiologically important and protective in neurodegenerative diseases (Singec and Snyder, 2008).

## Genetically engineered MSCs for therapeutic approaches in polyQ diseases

Since, MSCs fuse with damaged neurons, researchers have exploited their propensity; they have genetically modified them in order to deliver bioactive factors into cells at risk. This approach confers advantages over systemic administration or direct injection of e.g. genetically-modified viruses. Transplantation of genetically engineered MSCs (GE-MSCs) combines the advantages of cell and gene therapy. These cells have an intrinsic ability to express cell-trophic cytokines which promote neuronal growth, reduce release of free radicals and suppress inflammation (Olson et al., 2011b). At the same time, GE-MSCs may migrate to damaged brain areas and deliver their cargo directly to degenerating cells.

In the polyQ field, three attempts using GE-MSCs have been described. In HD, decreased levels of GDNF, NGF and BDNF in the brain are commonly observed. These neurotrophins contribute to growth and survival of neurons; therefore, their overexpression *in vivo* seems to be a logical strategy for the attenuation of the disease. Dey et al. transplanted GE-MSCs expressing BDNF or NGF into the striata of YAC128 transgenic mice and showed that transplanted mice presented less behavioral deficits. Mice receiving GE-MSCs had significantly less neuronal loss within the striatum surrounding the transplant area than control groups (Dey et al., 2010).

MSCs could be also used for RNAi transfer against polyQ proteins into damaged neurons. Olson et al. followed a similar strategy in order to suppress expression of mutant huntingtin (HTT) in neuronal cells. They generated MSCs expressing shRNA antisense to HTT and co-cultured them with cells expressing HTT142Q. Levels of HTT142Q were markedly reduced in neuronal cells indicating the direct communication and exchange of RNAi molecules between MSCs and HTT142Q expressing cells (Olson et al., 2011a).

Based on the property of MSCs to fuse with host PCs, Chen at al. tested a gene delivery approach for SCA1. Transplantation of GE-MSCs that overexpress chaperones attenuated disease pathogenesis in SCA1 mice. Binucleated Purkinje heterokaryons were detected and successfully expressed the modifier genes *in vivo*. Researchers also observed decreased number of nuclear inclusions containing polyQ ATXN1 aggregates and increase in the number of surviving Purkinje neurons (Chen et al., 2011). Taken together, these results indicate the potential use of GE-MSCs as gene/RNAi delivery vehicles in degenerating neurons. They also indicate the challenges that need to be addressed, such as the appropriate genetic modification method, number of cells, route and timing of transplantation and most importantly, selection of specific genes to overexpress in MSCs.

Viruses are commonly used to produce GE-MSCs. Despite their high transfection efficiency, nuclear transport of viral vectors and transgene expression may be impaired due to phosphorylation of the vector capsid and its subsequent degradation by the host proteasome machinery (Zhong et al., 2007). Therefore, non-viral methods may be more efficient for the generation and survival of GE-MSCs. The Sleeping Beauty transposon system is one of them; it has major advantages over viral vectors including high transgene expression, ease of handling and more importantly biosafety (Galla et al., 2011). In addition, it can be used for the genetic modification of MSCs and the generation of cell lines stably expressing a gene of interest (Petrakis et al., 2012a).

Concerning parameters such as the route of administration, number of cells to be transplanted and timing of the transplantation during disease progression, one should consider that MSCs should (a) reach the injured area and (b) survive for a substantial period of time in order to exert their therapeutic effect. MSCs are usually attracted by chemokines released by degenerating cells and activated microglia (Aarum et al., 2003). Following systemic administration, MSCs should cross the blood-brain-barrier in order to reach the brain. Therefore, a local administration of MSCs would be preferable considering the restrictions of extensive cell migration into the central nervous system. On the other hand, such a procedure is invasive and may damage the multilayer organization of neuronal tissue. Intrathecal administration of MSCs may represent an alternative and less invasive strategy (Matsuura et al., 2013). A critical point in such attempts is the survival of transplanted cells which may greatly influence their neuroprotective effect. Experimental evidence indicate that GE-MSCs are able to survive *in vivo* for a limited period of time compared to NPCs (Petrakis et al., unpublished data). Additionally, the effect of MSCs is more prominent when repeated doses of cells are administered (Zhang et al., 2011). These data indicate a limitation of GE-MSC transplantation; they also demonstrate the need to develop cell lines with a high survival potential that would not cause any adverse effect after *in vivo* transplantation.

GE-MSCs should also encode for genes that strongly suppress polyQ-induced neurodegeneration preferably at early stages of the disease. In the last years, several studies have described modifier genes that modulate cytotoxicity and aggregation of mutant proteins. Some of them, have a well-defined mode of action, suppressing the formation of toxic oligomers that are early formed in polyQ diseases and correlate with neuronal cell loss. For example, components of the ubiquitin-proteasome machinery upregulate proteasomal activity and their overexpression in damaged neurons may induce clearance of polyQ protein aggregates. At the molecular level, the interaction of polyQ ATXN1 with proteins like PUM1 or RBM17 is protective in SCA1 models (Lim et al., 2008; Petrakis et al., 2012b). Therefore, MSC-mediated delivery of e.g. PUM1 or RBM17 genes in Purkinje cells might favor protective protein interactions, prohibit toxic interactions and inhibit neurodegeneration in SCA1.

## Conclusions

Stem cells are a valuable tool to face polyQ diseases. Different types of them have different modes of actions. iPSCs can give rise to patient-specific neurons; together with NPCs they can be used for cell replacement therapies. MSCs can be easily isolated and modified to overexpress polyQ proteins, serving as disease models for drug discovery. When transplanted *in vivo*, GE-MSCs tend to home into injury sites and fuse with damaged neurons, delivering protective molecules. These cells have also the ability to promote neuronal survival. Potentially, co-transplantation of iPSCs and GE-MSCs may promote the engraftment of patient-specific iPSC-derived neurons and the survival of endogenous neurons in damaged brain areas. We strongly believe that stem cells hold great promise for the development of novel cell-based therapies for polyQ diseases.

## Author contributions

ES, SP: wrote the manuscript, GK: edited the manuscript.

### Conflict of interest statement

The authors declare that the research was conducted in the absence of any commercial or financial relationships that could be construed as a potential conflict of interest.

## Abbreviations

polyQ: polyglutamine
HD: Huntington's disease
DRPLA: Dentatorubral-pallidoluysian atrophy
SBMA: Spinal and bulbar muscular atrophy
SCA: Spinocerebellar ataxia
PC: Purkinje cell
iPSC: Induced pluripotent stem cell
MSC: Mesenchymal stem cell
ATXN1: ataxin-1
YFP: Yellow Fluorescent Protein
ES: embryonic cells
MUSE: multilineage differentiating stress enduring cells
MSN: medium spiny neuron
NPC: neural precursor cell
NTF-MSCs: neurotrophic factors-secreting MSCs
QA: quinolinic acid
GE-MSCs: genetically engineered MSCs
HTT: huntingtin.
